# Comparison between 75-g and 100-g oral glucose tolerance tests using international association of diabetes and pregnancy study group one-step diagnostic threshold to detect gestational diabetes mellitus

**DOI:** 10.3389/fendo.2025.1512499

**Published:** 2025-10-15

**Authors:** Li-Li Zhou, Dong Liu, Hong-Qing Song, Yuan-Bo Wu, Wei-Bing Hu, Jian-Feng Wang, Jin-Sheng Wang, Chun-Yan Qi, Sa-Sa Liu

**Affiliations:** ^1^ Department of Clinical Laboratory, People’s Hospital of Tongchuan, Tongchuan, Shaanxi, China; ^2^ Department of Gynaecology and Obstetrics, People’s Hospital of Tongchuan, Tongchuan, Shaanxi, China; ^3^ Department of Clinical Laboratory, The First Affiliated Hospital of Xi’an Jiaotong University, Xi’an, Shaanxi, China

**Keywords:** diagnostic accuracy, gestational diabetes mellitus, oral glucose tolerance test, adverse outcome, screening strategy, glucose dose

## Abstract

**Background:**

The oral glucose tolerance test (OGTT) is the primary screening method for gestational diabetes mellitus (GDM), but global implementation criteria remain inconsistent.

**Methods:**

This retrospective study analyzed data from 3,907 pregnant women at Tongchuan People’s Hospital, including 1,925 in the 75g OGTT group (430 with GDM) and 1,982 in the 100g OGTT group (460 with GDM). A systematic comparison was conducted between the two groups regarding: blood glucose levels at each time point (0h, 1h, 2h);diagnostic rates, positive composition ratios of gestational diabetes mellitus, and risks of adverse maternal and neonatal outcomes based on the International Association of Diabetes and Pregnancy Study Groups (IADPSG) diagnostic criteria; Correlation analysis of blood glucose levels across time points; A glucose-level-adjusted continuous analysis to evaluate the dose-response relationship between dynamic glucose changes and adverse maternal and neonatal outcomes in the overall population.

**Results:**

The 100g group had significantly higher 1h and 2h blood glucose levels than the 75g group (*p* < 0.01);Under the IADPSG criteria, there were no significant differences in GDM detection rates, positive case characteristics, or maternal-neonatal outcomes between the two groups (*p* > 0.05);Blood glucose levels at different time points were correlated within each group, no glucose rise difference occurred between groups at 0-1h [Difference in slope (95% CI): 0.127 (-0.092 to 0.346), p>0.05]. However, from fasting to 2h, the 100g group showed a steeper rise than the 75g group [Difference in slope (95% CI):0.412 (0.244 to 0.580), p<0.05], and a slower decline between 1-2h [Difference in slope (95% CI):0.047 (0.010 to 0.084), p<0.05].Glucose-adjusted continuous analysis showed that blood glucose levels were mostly associated with adverse outcomes, with the strength of association gradually decreasing from fasting to 1h and 2h. Both groups exhibited similar trends, no significant differences in the risks of adverse outcomes (expressed as ORs) were observed between the 75g and 100g OGTT groups (all *p* > 0.05).

**Conclusion:**

Under the IADPSG criteria, no significant differences in diagnostic efficacy were observed between the 75g and 100g OGTT glucose loads for GDM. Standardizing screening strategies to improve clinical consistency is warranted.

## 1 Introduction

Gestational diabetes mellitus (GDM) is a metabolic disease characterized by impaired glucose metabolism and is first detected or diagnosed during pregnancy. Its incidence increases with lifestyle and dietary changes. The prevalence of GDM is estimated at 9.3–25.5% worldwide (1, 2) and 9.3–18.9% in China (3, 4). Studies (5–7) have shown that GDM is associated with an increased risk of multiple adverse outcomes for both mother and baby, including cesarean section, neonatal hypoglycemia, and neonatal hyperbilirubinemia.

GDM is mainly diagnosed using the oral glucose tolerance test (OGTT), for which there is still a lack of consensus (8–10). There are two main strategies recommended internationally: the one-step strategy (2-h 75-g OGTT), which is recommended by the International Association of Diabetes and Pregnancy Study Group (IADPSG) (11), and the two-step strategy (50-g glucose loading test and 3-h 100-g OGTT), which is recommended by the American College of Obstetricians and Gynecologists(ACOG) (12). In addition to the two methods mentioned, other screening strategies are being used in some countries and regions (13–17). In mainland China, the IADPSG one-step 2-h 75-g OGTT was recommended to diagnose GDM by the Obstetrics Association of the Chinese Medical Association in 2014 (18). However, the latest version of the “National Guide to Clinical Laboratory Procedures, 4th edition (2014)” (19) was recommended by the National Health Commission of the People’s Republic of China later in 2014. The procedure suggested a 100-g glucose dose to perform OGTT for pregnant women, but the blood collection time point and diagnostic threshold were not clear. As a result, some laboratories in mainland China, including Tongchuan People’s Hospital, used the IADPSG one-step approach and the corresponding diagnostic threshold value to screen GDM for pregnant women, and the glucose load was 100 g. Although international recommendations for OGTT methods are inconsistent and lack the support or recognition of authoritative guidelines, the application of OGTT still exists objectively today. Evaluating these methods may play a positive role in the improvement of GDM screening strategies.

This study employed a multidimensional analytical approach to systematically evaluate the following key metrics of 75g versus 100g OGTT: 1) Blood glucose levels at fasting (0h), 1h, and 2h post-load timepoints; 2) GDM screening performance based on IADPSG criteria, including diagnostic positivity rate, clinical characteristics of GDM population, and differential risks of adverse maternal-neonatal outcomes; 3) Correlation patterns of glucose values across different timepoints (0h, 1h, 2h); 4) Dose-effect relationship between dynamic glucose variations and adverse maternal-neonatal outcomes in the overall study population.

## 2 Materials and methods

### 2.1 Participant sources

OGTT data for GDM screening were available for 3,907 of 10,228 primiparas who gave birth in two districts of Tongchuan People’s Hospital. This retrospective study covers the period from January 1, 2017, to September 30, 2022. The timeframe was selected based on comprehensive considerations including data availability, quality, consistency in clinical practice, and group sample size balance, with the aim of enhancing the scientific rigor and result reliability of the study. All primiparas who gave birth at the hospital during this period were enrolled, and their data were retrospectively analyzed using electronic medical records. Data extraction took place from April 16 to April 23, 2023.According to the actual screening strategy adopted, participants were divided into the 75-g and 100-g OGTT groups. Among these, the 75-g glucose dose recommended by the IADPSG was used in OGTT between October 1, 2019, and September 30, 2022, in the central southern campus, and between September 18, 2018, and September 30, 2022, in the northern campus. The 100-g glucose dose recommended in the guidelines was used in the OGTT experiments on the southern campus area from January 1, 2017, to September 30, 2019, and on the northern campus from January 1, 2017, to September 17, 2018. Women with maternal diabetes mellitus before pregnancy, multiple births, chronic kidney disease, and related endocrine diseases, such as hyperpituitarism, hyperthyroidism, and adrenal hyperfunction, were excluded from the study. The electronic medical records in this study have clearly identified individuals who experienced vomiting, and we have verified and excluded all data from subjects who experienced vomiting through electronic medical record review.

All procedures performed in studies involving human participants were in accordance with the ethical standards of the institutional and/or national research committee and with the 1964 Declaration of Helsinki and its later amendments or comparable ethical standards. The study design was approved by the Ethics Committee of Tongchuan People’s Hospital (approval number: TCSRMYY2022-01-03-005). The requirement for written informed consent was waived owing to the retrospective nature of the study. This retrospective study was conducted according to the STrengthening the Reporting of OBservational studies in Epidemiology guidelines. When we obtained the data, we obtained the patients’ identifying information, including name, address, identification number, telephone number, clinical diagnosis and treatment information, various examination results, etc.; however, only age, sex, outcome, and treatment interventions are disclosed in the manuscript.

### 2.2 Main observation index

We obtained patient data from electronic medical records such as age, pregnancy duration at GDM screening and delivery, BMI at GDM screening and delivery and status of serum glucose management and treatment of GDM. Serum glucose levels at 0, 1 h, and 2 h time points during the 75-g and 100-g OGTT were analyzed. The correlations and regression lines for glucose levels (fasting vs. 1 h, fasting vs. 2 h, and 1 h vs. 2 h) were compared between the two groups. The GDM diagnosis rate and positive composition characteristics of the two groups were assessed using the IADPSG one-step diagnostic threshold. Further, 15 adverse maternal and 16 neonatal outcomes were evaluated. The 15 adverse maternal outcomes included abnormal fetal membranes, abnormal stage of labor, abnormal umbilical cord, abnormal amniotic fluid volume, placental abnormalities, cesarean section, cholestatic syndrome, dystocia, hypoproteinemia, perineal laceration, pregnancy-induced hypertension, poor uterine rejuvenation after childbirth, postpartum hemorrhage, and postpartum infection, as well as amniotic/chorionic abnormalities, induction of labor, postpartum fever, and postpartum anemia. The 16 adverse neonatal outcomes included abnormal fetal position, fetal distress, fetal growth restriction, low birth weight, large for gestational age, low Apgar score, macrosomia, neonatal cranial hematoma, neonatal asphyxia, neonatal hyperbilirubinemia, neonatal hypoglycemia, neonatal infection, neonatal respiratory distress syndrome, preterm delivery, small for gestational age, and stillbirth. These adverse outcomes are defined in **Supplementary Methods 1**.

### 2.3 GDM screening approach

GDM screening approaches were similar in the northern and southern regions of the hospital. Pregnant women maintained normal physical activity, a normal diet, and daily carbohydrate consumption of at least 150 g for 3 days before the test. Pregnant women fasted for 10–12 h on the day before OGTT (which was conducted no later than 9 am). During examination, the participants did not drink tea, drink coffee, smoke, or engage in strenuous exercise. OGTT was performed 2 h after ingesting a standard 75-g or 100-g glucose load.

### 2.4 Determination of serum glucose levels

Venous blood was collected in a procoagulant negative pressure tube, allowed to stand for 20 min, and centrifuged (3,000 rpm) for 5 min to separate the serum. The serum glucose level was detected using a Hitachi 008AS automatic biochemical analyzer (Toranomon, Minato-ku, Tokyo, Japan) in the south campus and a Hitachi 7,600 automatic analyzer (Toranomon) in the north campus. All procedures were completed within 2 h of blood collection. Hexokinase glucose detection reagents were produced by Ningbo Meikang Co., Zhejiang, China. The internal quality control data were controlled during the testing period. The external quality assessment data from the Shaanxi Provincial Clinical Laboratory Center and the Clinical Laboratory Center of the National Health Commission of China were qualified.

### 2.5 Diagnosis, management, and treatment of GDM

The diagnostic criteria for GDM in both groups were based on the 2010 IADPSG one-step screening method (11). Pregnant women were diagnosed with GDM if any of the following glucose thresholds were met: 0 h ≥5.1 mmol/L; 1 h ≥10.0 mmol/L; and 2 h ≥8.5 mmol/L. Pregnant women with GDM should undergo diet, exercise, and drug treatment according to the “Diagnosis and therapy guideline of pregnancy with diabetes mellitus (2014)” (20) (see **Supplementary Methods 2** for details).

### 2.6 Statistical analyses

Data analysis was performed using SPSS Statistics 20.0 (IBM Corp., Armonk, NY, USA) for statistical computations and GraphPad Prism 8.0 (GraphPad Software Inc., La Jolla, CA, USA) for scatter plot generation. Continuous variables were assessed for normality via the Shapiro-Wilk test, with normally distributed data presented as mean ± standard deviation (mean ± SD) and compared using independent samples t-tests. Categorical variables were expressed as frequency (percentage), analyzed by chi-square tests. For OGTT glucose levels across timepoints (0h, 1h, 2h), intergroup comparisons were supplemented with Pearson correlation analyses and scatter plots.Employing a stratified analytical approach, we systematically evaluated 15 maternal and 16 neonatal adverse outcomes. In the GDM-positive cohort: 1) Potential determinants were screened through univariate analysis; 2) Multivariable unconditional logistic regression adjusted for baseline characteristics (age, pre-pregnancy BMI, gestational weight gain) to quantify outcome risk differences; 3) Log-linear modeling examined outcome interactions, with variance inflation factors (VIF <5) confirming absence of multicollinearity. For the full cohort, binary logistic regression modeled OGTT glucose levels (continuous) against adverse outcomes (dichotomous) to characterize dose-response relationships, adjusting for identical covariates. All analyses rigorously accounted for GDM diagnostic criteria and confounders—particularly excessive gestational weight gain per National Academy of Medicine standards (21, 22). Effects are reported as odds ratios (ORs) and adjusted odds ratios (aORs) with 95% confidence intervals. Statistical significance for primary outcomes was defined as *p* < 0.05 (two-tailed α=0.05). (Detailed protocols: **Supplementary Methods 3**).

## 3 Results

### 3.1 Baseline characteristics of the study population

After applying the exclusion criteria, this study included 1,925 pregnant women (430 with GDM) in the 75-g OGTT group and 1,982 pregnant women (460 with GDM) in the 100-g OGTT group. Maternal age, pregnancy duration at GDM, body mass index (BMI), and incidences of other abnormalities were calculated (**Table 1**). No significant difference was noted in these characteristics between the groups (*p* > 0.05). Similarly, pregnancy duration and BMI at the time of delivery showed no significant differences (*p* > 0.05; **Table 1**). There was no significant difference in serum glucose control among GDM-positive people between the groups (*p* > 0.05; **Supplementary Table 1**).

**Table 1:** Intergroup differences in the baseline characteristics of the study population.

Characteristics	75-g OGTT, mean ± SD (n = 1,925)	100-g OGTT, mean ± SD (n = 1,982)	T/(χ^2^)	*P*
Maternal age (years)	29.69 ± 4.02	29.55 ± 3.98	−1.141	0.254
Pregnancy duration at GDM screening (weeks)	26.02 ± 1.30	26.03 ± 1.34	0.246	0.806
BMI at GDM screening (kg/m^2^)	23.37 ± 2.79	23.38 ± 2.81	0.124	0.902
Pregnancy duration of delivery (weeks)	39.07 ± 1.76	39.10 ± 2.02	0.525	0.599
BMI at the time of delivery (kg/m^2^)	29.20 ± 2.63	29.32 ± 2.56	1.496	0.135
Incidence of other abnormalities^*^ [% (n/n)]	1.87 (36/1,925)	2.12 (42/1,982)	0.309	0.648

t/χ^2^: Student’s *t*-test was performed for continuous variables and chi-square test for count data. ^*^Other abnormalities included traumatic fractures, pregnancy with cholecystitis, pregnancy with pancreatitis, pregnancy with chronic nephritis, pregnancy with tuberculosis, and pregnancy with heart disease. The rate is expressed as a proportion (%; number of positives/total). The chi-square test was performed for the comparison of rates. BMI, body mass index; GDM, gestational diabetes mellitus; OGTT, oral glucose tolerance test.

### 3.2 Comparison of serum glucose levels between the groups

There was no significant difference in fasting glucose levels between the two groups (*p* > 0.05). The serum glucose levels at 1 h and 2 h after oral glucose were significantly lower in the 75-g group than in the 100-g group (*p* < 0.05), as shown in **Table 2**.

**Table 2:** Intergroup comparison of serum glucose levels at each time point.

Time point	75-g OGTT, serum glucose mean ± SD, mmol/L (n = 1,925)	100-g OGTT, serum glucose mean ± SD, mmol/L (n = 1,982)	*T*	*P*
Fasting	4.68 ± 0.49	4.69 ± 0.48	0.811	0.417
1 h	7.52 ± 1.90	7.72 ± 1.84	3.214	0.001
2 h	6.58 ± 1.50	6.75 ± 1.38	3.661	0.000

OGTT, oral glucose tolerance test.

### 3.3 Comparison of GDM diagnostic rates, positive composition ratio, and adverse outcomes between groups

Using IADPSG one-step criteria, no significant differences were observed in GDM diagnostic rates or positive case characteristics between groups (*p* > 0.05; **Table 3**). Similarly, maternal and neonatal adverse outcomes showed no significant differences (*p* > 0.05; **Tables 4**, **5**). Given potential confounding by age, gestational age, BMI trajectory, and post-diagnosis interventions, we performed full covariate adjustment (**Supplementary Table 2**). Logistic regression analysis using the 75g group as reference demonstrated that the 100g group’s risk profile for adverse outcomes (expressed as aORs) remained stable before versus after adjustment (*p* > 0.05; **Tables 4**, **5**). In the GDM-negative population, there were no significant differences in the risks of adverse outcomes between the 75g and 100g oral glucose tolerance tests, except for the “other” outcomes category (*p* > 0.05). Among those screened and diagnosed with GDM who received corresponding management, the risks of adverse pregnancy outcomes showed no significant difference compared to the GDM-negative group, except for cesarean delivery (*p* > 0.05). In contrast, the screened group demonstrated a statistically significant reduction in the risk of major adverse pregnancy outcomes compared to the unscreened group (*p* < 0.05); for detailed results, please refer to **Supplementary Table 3**.

**Table 3:** Intergroup comparisons of the GDM diagnostic rate and positive composition ratio [%, (n/n)].

Positive modes (mmol/L)	75-g OGTT (%) (n = 430)	100-g OGTT (%) (n = 460)	*χ^2^*	*P*
Only fasting ≥5.1	42.33 (182/430)	37.17 (171/460)	2.465	0.131
Only 1 h ≥10.0	11.63 (50/430)	12.39 (57/460)	0.122	0.758
Only 2 h ≥ 8.5	11.16 (48/430)	12.83 (59/460)	0.581	0.471
Fasting ≥ 5.1 and 1 h ≥ 10.0	7.21 (31/430)	8.70 (40/460)	0.669	0.458
Fasting ≥ 5.1 and 2 h ≥ 8.5	5.35 (23/430)	3.70 (17/460)	1.415	0.259
1 h ≥ 10.0 and 2 h ≥ 8.5	8.14 (35/430)	11.52 (53/460)	2.853	0.093
Fasting ≥ 5.1, 1 h ≥ 10.0, and 2 h ≥ 8.5	14.19 (61/430)	13.70 (63/460)	0.045	0.847
Total positive rate of GDM	22.34 (430/1,925)	23.21 (460/1,982)	0.421	0.517

GDM, gestational diabetes mellitus; OGTT, oral glucose tolerance test.

**Table 4:** Intergroup comparison of maternal outcomes.

Maternal outcomes	Unadjusted		Adjusted^※^	
	OR (95% CI)	*P*	aOR (95% CI)	*P*
Abnormal fetal membranes	0.97 (0.68–1.40)	0.885	0.96 (0.67–1.40)	0.884
Abnormal stage of labor	0.93 (0.30–2.92)	0.906	0.97 (0.24–3.12)	0.901
Abnormal umbilical cord	0.97 (0.73–1.29)	0.828	0.98 (0.69–1.30)	0.830
Amniotic fluid volume abnormality	1.11 (0.69–1.79)	0.670	1.10 (0.41–1.79)	0.528
Cesarean section	0.86 (0.66–1.12)	0.260	0.89 (0.75–1.19)	0.301
Cholestatic syndrome	1.25 (0.52–3.00)	0.613	1.28 (0.48–3.01)	0.608
Dystocia	1.24 (0.64–2.41)	0.529	1.27 (0.79–2.45)	0.595
Hypoproteinemia	1.25 (0.52–3.00)	0.613	1.29 (0.68–3.02)	0.686
Perineal laceration	1.04 (0.76–1.42)	0.803	1.09 (0.69–1.48)	0.801
Pregnancy-induced hypertension	0.96 (0.57–1.62)	0.889	1.01 (0.84–1.19)	0.885
Placental abnormalities	1.28 (0.58–2.83)	0.535	1.34 (0.85–1.89)	0.517
Poor postpartum uterine rejuvenation	1.08 (0.58–1.99)	0.808	1.05 (0.62–1.94)	0.843
Postpartum hemorrhage	1.69 (0.74–3.86)	0.216	1.79 (0.91–2.95)	0.249
Postpartum infection	1.25 (0.52–3.00)	0.613	1.27 (0.48–3.01)	0.608
Other^#^	1.11 (0.49–2.50)	0.805	1.19 (0.71–2.57)	0.884

^#^Other conditions included amniotic/chorionic abnormalities, induction of labor, postpartum fever, and postpartum anemia. ^※^Adjusted for GDM and covariates associated with non-adherence: maternal age, BMI, pregnancy history, insulin treatment, and chronic hypertension. OR, odds ratio; CI, confidence interval; OGTT, oral glucose tolerance test.

**Table 5:** Intergroup comparison of neonatal outcomes in progeny.

Neonatal outcomes	Unadjusted		Adjusted^※^	
	OR (95% CI)	*P*	aOR (95% CI)	*P*
Abnormal fetal position	1.10 (0.80–1.51)	0.560	1.11 (0.82–1.71)	0.561
Fetal distress	1.50 (0.49–4.64)	0.477	1.59 (0.21–4.75)	0.479
Fetal growth restriction	1.28 (0.58–2.83)	0.535	1.27 (0.55–2.20)	0.553
Low birth weight	1.07 (0.39–2.98)	0.898	1.05 (0.32–2.67)	0.891
Large for gestational age	0.93 (0.39–2.27)	0.879	1.00 (0.31–2.29)	0.892
Low Apgar score	1.70 (0.56–5.10)	0.347	1.81 (0.67–5.55)	0.374
Macrosomia	1.17 (0.71–1.92)	0.547	1.21 (0.74–1.93)	0.585
Neonatal cranial hematoma	1.15 (0.68–1.92)	0.607	1.14 (0.63–1.29)	0.603
Neonatal asphyxia	1.31 (0.41–4.17)	0.644	1.32 (0.84–4.21)	0.669
Neonatal hyperbilirubinemia	0.99 (0.72–1.36)	0.957	0.91 (0.65–1.01)	0.929
Neonatal hypoglycemia	1.22 (0.53–2.82)	0.639	1.29 (0.17–2.90)	0.801
Neonatal infection	1.02 (0.73–1.43)	0.906	1.05 (0.76–1.55)	0.959
Neonatal respiratory distress syndrome	1.25 (0.43–3.64)	0.681	1.16 (0.06–3.24)	0.620
Preterm delivery	1.38 (0.81–2.33)	0.237	1.41 (0.45–2.39)	0.298
Small for gestational age	1.41 (0.39–5.02)	0.598	1.61 (0.36–5.25)	0.601
Stillbirth	1.17 (0.31–4.39)	0.816	1.19 (0.35–4.41)	0.857

^※^Adjusted for GDM and covariates associated with non-adherence: maternal age, BMI, pregnancy history, insulin treatment, and chronic hypertension. OR, odds ratio; aOR, adjusted odds ratio, CI, confidence interval; OGTT, oral glucose tolerance test.

### 3.4 Intergroup analysis of glycemic correlations

**Figure 1:**
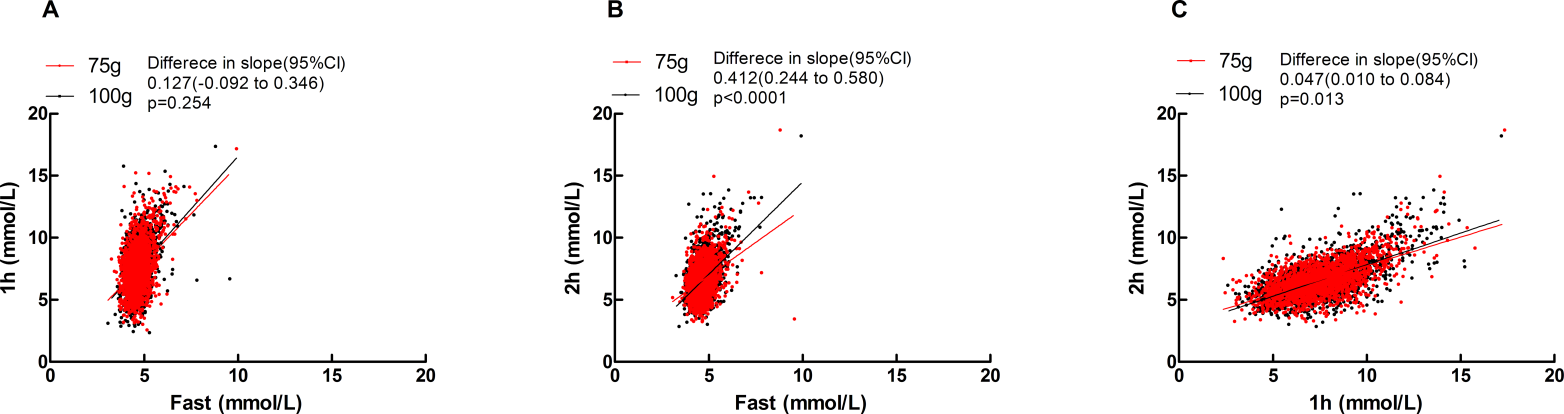
Scatters of fast Vs 1h, fast Vs 2h, 1h Vs 2h in two groups. **(A)**fast Vs 1h; **(B)** fast Vs 2h; **(C)** 1h Vs 2h. Solid lines represent regression fits for each group (red: 75 g group; black: 100 g group). Difference in slope was defined as the slope of the 100 g group minus that of the 75 g group.

Significant positive correlations were observed between fasting vs. 1h, fasting vs. 2 h, and 1 h vs. 2 h blood glucose levels in two groups (see **Supplementary Table 4**). The effects of different glucose loads (75-g vs. 100-g) on glycemic kinetics demonstrated distinct phase-specific variations: During the fasting-to-1h phase, the rate of glucose elevation (slope) showed no statistically significant difference between the two groups [Difference in slope (95% CI): 0.127 (-0.092 to 0.346), p=0.254]; in the fasting-to-2 h phase, the 100-g group exhibited a significantly higher glucose elevation rate than the 75-g group [Difference in slope (95% CI): 0.412 (0.244 to 0.580), p<0.0001]; during the 1h-to-2 h phase, glucose decline occurred significantly more slowly in the 100-g group [Difference in slope (95% CI): 0.047 (0.010 to 0.084), p=0.013], see **Figure 1**.

### 3.5 Analysis of the continuous dose-response relationship between blood glucose levels and adverse outcomes in two groups

After adjusting for potential confounders, no significant differences were observed in the incidence of any adverse outcomes between the two groups (all *p >*0.05; **Tables 6**, **7**). The effects of glucose levels varied by timepoints.For example, for cesarean delivery risk, each 1 mmol/L increase in fasting glucose was associated with a 27.5% significantly higher risk (aOR=1.275, 95%CI:1.084-1.501, *p=*0.003), while 1-h postprandial glucose showed a 5.1% increased risk per 1 mmol/L (aOR=1.051, 95%CI:1.004-1.100, *p=*0.032), with no significant effect of 2-h glucose (*p* = 0.649); for macrosomia risk, although neither fasting (aOR=1.33, 95%CI:0.98-1.81, *p=*0.072), 1-h (aOR=0.99) nor 2-h glucose (aOR=0.97) reached statistical significance, the effect size and upper 95%CI limit of fasting glucose suggested potential clinical relevance. Detailed results for other adverse outcomes are shown in **Tables 6** and **Table 7**.

**Table 6:** Dose adjusted continuous analysis of the maternal outcomes (75g, n=1,925; 100g, n=1,982).

Outcomes	Variable	aOR (95% CI)	*P*
Cesarean section	groups	0.92 (0.81-1.05)	0.210
	fasting	1.28 (1.08-1.50)	0.003
	1hr	1.05 (1.00-1.10)	0.032
	2hr	1.01 (0.96-1.08)	0.649
Abnormal fetal membranes	groups	1.03 (0.87-1.23)	0.729
	fasting	1.01 (0.82-1.26)	0.904
	1hr	0.96 (0.90-1.02)	0.206
	2hr	1.08 (1.00-1.17)	0.054
Placental abnormalities	groups	1.03 (0.73-1.45)	0.883
	fasting	0.71 (0.45-1.12)	0.137
	1hr	1.07 (0.95-1.21)	0.259
	2hr	0.95 (0.81-1.11)	0.500
Abnormal umbilical cord	groups	0.93 (0.81-1.06)	0.272
	fasting	1.04 (0.88-1.24)	0.656
	1hr	0.99 (0.95-1.04)	0.779
	2hr	0.98 (0.92-1.04)	0.453
Amniotic fluid volume abnormality	groups	0.73 (0.55-0.97)	0.029
	fasting	1.21 (0.87-1.68)	0.256
	1hr	1.02 (0.93-1.13)	0.638
	2hr	1.01 (0.89-1.14)	0.931
Abnormal stage of labor	groups	0.33 (0.06-1.82)	0.204
	fasting	2.44 (0.71-8.38)	0.156
	1hr	0.70 (0.41-1.20)	0.192
	2hr	0.90 (0.45-1.82)	0.774
Dystocia	groups	0.99 (0.74-1.32)	0.938
	fasting	0.90 (0.62-1.30)	0.565
	1hr	0.91 (0.83-1.01)	0.084
	2hr	1.10 (0.96-1.25)	0.162
Pregnancy-induced hypertension	groups	1.65 (1.12-2.42)	0.011
	fasting	0.96 (0.58-1.59)	0.885
	1hr	1.00 (0.87-1.14)	0.969
	2hr	0.94 (0.79-1.12)	0.486
Cholestatic syndrome	groups	1.08 (0.57-2.06)	0.814
	fasting	0.55 (0.24-1.25)	0.154
	1hr	1.26 (1.02-1.57)	0.036
	2hr	0.99 (0.75-1.31)	0.939
Perineal laceration	groups	0.91 (0.78-1.06)	0.211
	fasting	0.92 (0.76-1.12)	0.391
	1hr	0.94 (0.89-0.99)	0.017
	2hr	1.04 (0.97-1.12)	0.262
Postpartum hemorrhage	groups	1.06 (0.45-2.51)	0.887
	fasting	0.44 (0.15-1.29)	0.135
	1hr	1.28 (0.97-1.70)	0.086
	2hr	1.02 (0.71-1.46)	0.934
Postpartum infection	groups	0.86 (0.37-2.01)	0.727
	fasting	1.43 (0.62-3.28)	0.403
	1hr	0.86 (0.64-1.15)	0.313
	2hr	1.25 (0.88-1.77)	0.221
Poor postpartum uterine rejuvenation	groups	1.14 (0.87-1.50)	0.348
	fasting	0.83 (0.58-1.19)	0.304
	1hr	1.07 (0.97-1.18)	0.173
	2hr	0.91 (0.80-1.04)	0.151
Hypoproteinemia	groups	0.34 (0.19-0.61)	0.000
	fasting	0.87 (0.46-1.66)	0.673
	1hr	0.89 (0.74-1.06)	0.192
	2hr	1.08 (0.85-1.37)	0.521

Adjusted for GDM and covariates associated with non-adherence: maternal age, BMI, pregnancy history, insulin treatment, and chronic hypertension. aOR, adjusted odds ratio; CI, confidence interval; OGTT, oral glucose tolerance test.

**Table 7:** Dose adjusted continuous analysis of the neonatal outcomes in progeny (75g, n=1,925; 100g, n=1,982).

Outcomes	Variable	aOR (95% CI)	*P*
Fetal distress	groups	0.59 (0.24-1.44)	0.242
	fasting	1.19 (0.43-3.31)	0.735
	1hr	1.15 (0.86-1.55)	0.335
	2hr	0.79 (0.53-1.17)	0.231
Abnormal fetal position	groups	1.84 (1.56-2.17)	0.000
	fasting	1.23 (1.01-1.51)	0.041
	1hr	0.92 (0.87-0.97)	0.002
	2hr	1.03 (0.96-1.11)	0.372
Stillbirth	groups	1.01 (0.52-1.99)	0.967
	fasting	0.53 (0.23-1.26)	0.152
	1hr	1.05 (0.83-1.33)	0.671
	2hr	1.20 (0.90-1.60)	0.221
Preterm infant	groups	0.86 (0.61-1.21)	0.392
	fasting	0.98 (0.65-1.48)	0.929
	1hr	1.11 (0.99-1.25)	0.073
	2hr	0.96 (0.82-1.12)	0.598
Small for gestational age (SGA)	groups	1.25 (0.27-5.72)	0.773
	fasting	1.18 (0.22-6.28)	0.847
	1hr	1.11 (0.66-1.88)	0.688
	2hr	1.04 (0.55-1.97)	0.904
Large for gestational age (LGA)	groups	1.32 (0.88-1.96)	0.177
	fasting	1.05 (0.64-1.70)	0.857
	1hr	1.11 (0.97-1.28)	0.119
	2hr	0.96 (0.81-1.15)	0.661
Low birth weight infant	groups	0.80 (0.50-1.30)	0.375
	fasting	0.81 (0.44-1.48)	0.491
	1hr	1.09 (0.92-1.28)	0.334
	2hr	0.98 (0.79-1.22)	0.886
Macrosomia	groups	0.83 (0.64-1.09)	0.183
	fasting	1.33 (0.98-1.81)	0.072
	1hr	0.99 (0.90-1.09)	0.862
	2hr	0.97 (0.86-1.10)	0.641
Neonatal hypoglycemia	groups	0.77 (0.50-1.18)	0.230
	fasting	1.05 (0.61-1.82)	0.864
	1hr	1.05 (0.90-1.22)	0.551
	2hr	0.88 (0.71-1.07)	0.199
Neonatal hyperbilirubinemia	groups	1.00 (0.86-1.17)	0.976
	fasting	1.08 (0.89-1.31)	0.446
	1hr	1.00 (0.95-1.06)	0.968
	2hr	0.99 (0.92-1.06)	0.769
Neonatal asphyxia	groups	1.23 (0.45-3.35)	0.683
	fasting	1.70 (0.68-4.26)	0.260
	1hr	1.21 (0.86-1.69)	0.269
	2hr	0.78 (0.51-1.20)	0.254
Neonatal infection	groups	1.09 (0.92-1.29)	0.314
	fasting	1.31 (1.07-1.61)	0.009
	1hr	1.00 (0.94-1.06)	0.988
	2hr	0.97 (0.90-1.05)	0.401
Low Apgar score	groups	0.93 (0.41-2.14)	0.872
	fasting	0.98 (0.38-2.52)	0.969
	1hr	1.19 (0.90-1.57)	0.232
	2hr	1.00 (0.70-1.42)	0.989
Neonatal cephalohematoma	groups	1.09 (0.84-1.43)	0.519
	fasting	0.73 (0.52-1.04)	0.080
	1hr	0.99 (0.91-1.09)	0.904
	2hr	1.12 (0.99-1.26)	0.073
Fetal growth restriction (FGR)	groups	1.64 (0.59-4.54)	0.340
	fasting	0.98 (0.27-3.56)	0.969
	1hr	0.88 (0.62-1.25)	0.466
	2hr	1.14 (0.73-1.79)	0.571
Neonatal respiratory distress syndrome (NRDS)	groups	0.59 (0.24-1.44)	0.242
	fasting	1.19 (0.43-3.31)	0.735
	1hr	1.15 (0.86-1.55)	0.335
	2hr	0.79 (0.53-1.17)	0.231

Adjusted for GDM and covariates associated with non-adherence: maternal age, BMI, pregnancy history, insulin treatment, and chronic hypertension. aOR, adjusted odds ratio; CI, confidence interval; OGTT, oral glucose tolerance test.

## 4 Discussion

The international controversy regarding the standardization of GDM screening persists, primarily manifested in three aspects: First, fundamental discrepancies exist in international guidelines—the IADPSG recommends the one-step 75g approach, while the ACOG advocates the two-step 50g+100g method, with significant differences in key parameters including glucose load, blood sampling timepoints, and diagnostic thresholds (13, 23, 24). Second, global implementation standards demonstrate regional variations: some countries rely solely on 2h glucose values while others incorporate both 1h and 2h measurements (14); within the United States alone, cutoff values for the 50g screening test vary between 7.2, 7.5, and 7.8 mmol/L across different states (23); and mainland China, while adopting the NDDG standard framework, employs IADPSG diagnostic cutoffs (13). Third, screening strategy selection is further influenced by multiple factors including regional epidemiological characteristics, healthcare resource allocation, and cultural acceptance (13, 14, 23). This global inconsistency in standards not only fuels diagnostic controversies regarding over- or under-diagnosis of GDM, but also severely compromises the comparability of epidemiological data, underscoring the urgent need for establishing internationally unified screening criteria. Against this backdrop, this study focuses specifically on evaluating differences between 75g and 100g glucose loads in OGTT-based GDM screening, aiming to provide evidence-based support for developing standardized protocols.

This study systematically evaluated the diagnostic performance of the 100g 2h OGTT for GDM screening and pregnancy outcome prediction, using the one-step 75g 2h OGTT recommended by the IADPSG as the reference standard. The results demonstrated that although the 100g group showed significantly higher postprandial glucose levels at 1h and 2h timepoints compared to the 75g group (*p* < 0.05, **Table 2**), no statistically significant differences were observed between the two groups in fasting glucose levels, GDM diagnosis rates, or clinical characteristics of GDM-positive individuals (*p*>0.05, **Table 3**). These findings likely reflect the physiological mechanisms of glucose homeostasis maintained through multi-organ coordination, including hepatic glucose metabolism regulation, compensatory insulin secretion, and peripheral tissue glucose uptake (24, 25). This suggests that the difference in glucose loads between 75-100g may not exceed the threshold required to disrupt the body’s compensatory balance, thereby failing to induce significant metabolic disturbances. These results provide important physiological evidence for selecting appropriate OGTT glucose loads in clinical practice.

Current evidence demonstrates that clinical management of GDM exerts greater influence on pregnancy outcomes than screening method selection (26, 27). Our study revealed consistent clinical interventions between the two GDM groups, with potential confounders controlled through restriction to primiparous women and adjustment for covariates including BMI trajectory. Notably, GDM and excessive gestational weight gain exhibited significant interaction effects on both cesarean delivery rate and gestational hypertension incidence (*p* < 0.05; **Supplementary Table 2**). After comprehensive adjustment, both groups showed comparable risks of adverse outcomes (*p*>0.05, **Tables 4**, **5**). In the GDM-negative population, no statistically significant differences were observed in the risks of adverse outcomes between the 75g and 100g oral glucose tolerance tests, except for the “other” outcomes category (**Supplementary Table 3**). This indicates that under the IADPSG criteria, the two OGTT loads have comparable predictive value. The observed difference within the “other” category may be due to the limited sample size, and further validation in larger studies is warranted.

Under a unified diagnostic criterion—that is, using identical glucose thresholds and cut-off values—the volume of the OGTT glucose load (75g versus 100g) does not significantly impact the diagnostic efficacy for GDM or alter the risks associated with adverse pregnancy outcomes. This result aligns with existing literature emphasizing the central importance of diagnostic thresholds (reference 14). Moreover, among those diagnosed with GDM through screening and subsequently managed, the risks for most adverse outcomes did not differ significantly from those in the GDM-negative population (**Supplementary Table 3**), highlighting the effectiveness of systematic GDM management. However, the higher rate of cesarean delivery observed in the GDM-positive group suggests that GDM may itself be an independent risk factor for cesarean section. The elevated risk of adverse outcomes in the unscreened group (**Supplementary Table 3**) further underscores the clinical importance of implementing OGTT screening and appropriate GDM management.

Dynamic glycemic correlation analysis revealed significant yet modest time-dependent correlations (fasting→1h→2h) within both 75g and 100g glucose load groups (all R²=0.138-0.413, p<0.0001; **Supplementary Table 4**). These findings indicate that: (1) Fasting glucose levels, serving as metabolic baselines, partially predict subsequent glycemic responses but explain limited variation (≤24.0%); (2) The fasting vs. 2h glucose association was stronger under 100g loading (75g R²=0.138 vs. 100g R²=0.240), suggesting high-dose amplification of inter-individual baseline variations with potential implications for diabetes risk stratification; (3) Collinear effects between fasting and dynamic glucose levels (e.g., each 1 mmol/L fasting increase caused 0.412 mmol/L additional 2h glucose elevation specifically in 100g group) underscore the necessity of baseline adjustment in clinical trials, which could otherwise mask true intervention effects.

**Figure 1** demonstrated comparable fasting-to-1h glucose elevation rates between 75g and 100g glucose loads (no dose-dependent difference in early-phase response). The 100g group exhibited significantly accelerated glucose rise during fasting-to-2h phase (indicating dose-amplified late-phase hyperglycemia) and attenuated glucose decline at 1h-to-2h phase. Collectively, 100g loading altered glucose metabolism through enhanced late-phase glycemic surge and prolonged hyperglycemia, whereas 75g loading better maintained glucose homeostasis. These differential responses reflected more stable/efficient physiological regulation of 75g glucose.

Given the absence of statistically significant differences in outcome risks among women diagnosed with GDM based on diagnostic cutoff values, we conducted an in-depth analysis using binary logistic regression models. In these models, the occurrence of adverse outcomes served as the dichotomous dependent variable, while glucose levels at each time point were included as continuous independent variables. The analysis incorporated adjustments for potential confounding factors, including interactions between glucose levels at different time points, to systematically evaluate the risk of adverse outcomes in the entire study population across both groups. The results demonstrated that although glucose levels at various time points showed correlations with most adverse outcomes, with varying degrees of association for different outcomes, none of the adverse outcome rates exhibited statistically significant differences between the two groups (all *p* > 0.05; **Tables 6**, **7**). These findings provide robust evidence that the glucose load is not a primary determinant influencing the occurrence of adverse outcomes.

The incidence of adverse outcomes in this study differed from those in other studies; for example, the incidences of hypoproteinemia in the 75-g and 100-g OGTT groups in our study were 2.09% (9/430) and 2.61% (12/460), respectively. Yuen et al. (28) reported that the incidence of hypoproteinemia was 4.6%. However, the incidence of macrosomia between the two groups in our study was 6.98% (30/430) and 8.04% (37/460), respectively. Moreover, Niroomand et al. (29) reported the incidence of macrosomia as 4.5%. These differences may be due to the occurrence of GDM influenced by region, socioeconomic status, and nutritional status (1–4), not related to the OGTT glucose dose.

All data in this study were collected from two campuses in Tongchuan People’s Hospital. The total number of primiparas in this region from 2017 to 2022 was 20,042 (http://www.tongchuan.gov.cn/), of whom 6,427 were at Tongchuan People’s Hospital. Ultimately, a total of 3,907 primiparas (19.49%) were included in this study. Therefore, this research provides a good representation of this region. Moreover, the total numbers of adverse outcomes of pregnant women and newborns in this study were 15 and 16, respectively, more than those included in many other similar studies (27, 29).

This study has several limitations. Ideally, both the IADPSG and C&C criteria should have been applied for cross-analysis of the two groups. However, due to the retrospective design, the historical 100g OGTT tests did not include the 3-hour glucose measurement. Moreover, the 100g OGTT was intended to be performed only after a positive 50g GCT preliminary screening—a test not routinely conducted at our institution—making related data unavailable. Similarly, applying the C&C criteria was not feasible for the 75g OGTT group due to the lack of 3-hour glucose values. Given considerations of data accessibility and reliability, the IADPSG criteria (i.e., the 75g OGTT and its diagnostic thresholds) were uniformly used in this analysis. Additionally, information on the management and treatment of gestational diabetes mellitus (GDM) could only be obtained through retrospective medical record review, and statistical methods were employed to minimize inaccuracies. Nonetheless, lifestyle factors such as alcohol consumption, dietary quality, physical activity level, as well as socioeconomic indicators beyond education, were generally not systematically documented in medical records. This may have resulted in residual confounding and might have influenced the outcomes. Furthermore, since December 2019, the COVID-19 pandemic has affected both GDM screening and post-diagnosis management (30). This factor was not assessed in the present study and may also represent a potential source of interference.

In summary, under the IADPSG criteria, our study found no significant differences in GDM detection rates or adverse pregnancy outcomes between the 75-g and 100-g OGTT protocols. These results suggest that the two loads have comparable diagnostic and prognostic performance; however, a formal equivalence or non-inferiority trial is ultimately required to confirm true equivalence. To enhance clinical consistency and comparability across practices, we recommend that countries or regions move toward adopting a unified OGTT glucose load. The development of such a standardized screening strategy should be informed by multidisciplinary expertise, encompassing clinical, laboratory, health economic, and sociological perspectives.

## Data Availability

The original contributions presented in the study are included in the article/**Supplementary Material**. Further inquiries can be directed to the corresponding author.
