# Supplementary material for: Comparison between 75-g and 100-g oral glucose tolerance tests using international association of diabetes and pregnancy study group one-step diagnostic threshold to detect gestational diabetes mellitus
Source: Front Endocrinol (Lausanne). 2025 Oct 15;16:1512499. doi: 10.3389/fendo.2025.1512499 (PMC12568363; doi:10.3389/fendo.2025.1512499)
Supplement: Supplementary file 1 [file DataSheet1.docx]

***Supplementary Material***

**1 Supplementary Methods**

**1.1 Definition of Major Adverse Outcomes Between Pregnant Women and Newborns with and without GDM Screening Data**

**1.1.1 Definition of Adverse Maternal Outcomes**

1. Abnormal fetal membranes: refers to premature rupture of the membranes.
2. Abnormal stage of labor: this encompasses various conditions such as prolonged latency, prolonged active labor, stagnation of active labor, stagnation of the second stage of labor, delayed descent of the fetal head, and stagnation of fetal head descent.
3. Abnormal umbilical cord: umbilical cord abnormalities include excessively long or short cords, cord entanglement, knotting, twisting, prolapse, and abnormal cord insertion.
4. Amniotic fluid volume abnormality: this includes oligohydramnios (amniotic fluid volume <300 mL in the third trimester) and polyhydramnios (amniotic fluid volume >2,000 mL during any period of pregnancy).
5. Cesarean section: surgical delivery of a fetus through an abdominal incision at or before full term.
6. Intrahepatic cholestasis of pregnancy: characterized by uninduced skin pruritus and serum bile acids ≥11 µmol/L.
7. Labor dystocia: also known as abnormal labor, characterized by slow or prolonged labor.
8. Hypoproteinemia: plasma total protein <6 g/L.
9. Perineal laceration: refers to tears in the perineum, classified into four degrees; this manuscript focuses on degrees III and IV.
10. Pregnancy-induced hypertension: defined as systolic blood pressure ≥140 mmHg and diastolic blood pressure ≥90 mmHg.
11. Placental abnormalities: this includes placenta previa, placenta accreta, placental increta, and placental abruption.
12. Poor postpartum uterine rejuvenation: the uterus fails to return to a non-pregnant state 6 weeks after delivery.
13. Postpartum hemorrhage: excessive blood loss within 24 h after delivery, with blood loss during vaginal delivery ≥500 mL or cesarean delivery ≥1,000 mL, or with signs of low blood volume after blood loss.
14. Postpartum infection: infections of the genital tract and surrounding tissues from the onset of labor or rupture of membranes until 42 days postpartum.
15. Other conditions include amniotic/chorionic abnormalities, induction of labor, postpartum fever, and postpartum anemia.

# **1.1.2 Definition of Adverse Infant Outcomes**

1. Abnormal fetal position: refers to the abnormal positioning of the fetus in the uterus after 30 weeks of pregnancy, including breech, transverse, occipital, and facial positions.
2. Fetal distress: defined by a rapidly deteriorating or abnormal cardiotocographic pattern and/or fetal scalp pH <7.20.
3. Fetal growth restriction: birth weight of the fetus <2,500 g after 37 weeks of gestation.
4. Low birth weight: birth weight of the baby <2,500 g.
5. Large for gestational age: defined as an estimated fetal weight >90th percentile for gestational age.
6. Low Apgar score: score <7 at 1 min, used globally to assess the vitality of newborns in their first minutes of life.
7. Macrosomia: defined as birth weight >4,000 g.
8. Neonatal cranial hematoma: injurious hemorrhage during birth of the newborn, diagnosed definitively by head magnetic resonance imaging.
9. Neonatal asphyxia: characterized by prenatal risk factors, Apgar score <7 at 1 or 5 min, umbilical arterial blood pH < 7.15, with exclusion of other causes of low Apgar score.
10. Neonatal hyperbilirubinemia: bilirubin level >85 μmol/L (5 mg/dL).
11. Neonatal hypoglycemia: blood sugar levels <2.2 mmol/L within 72 h of birth.
12. Neonatal infection: inflammatory diseases caused by various viruses or bacteria.
13. Neonatal respiratory distress syndrome: respiratory distress within 24 h of birth, responding well to treatment with surfactant and pulmonary re-expansion.
14. Preterm delivery: birth occurring prior to 37 weeks’ gestation.
15. Small for gestational age: birth weight less than the 10th percentile for the same gestational age.
16. Stillbirth: occurs when a fetus dies in the uterus.

**1.2 Management and Treatment of GDM**

According to the “Diagnosis and Therapy Guideline of Pregnancy with Diabetes Mellitus (2014)” GDM patients should aim to control their serum glucose levels during pregnancy to ≤5.3 mmol/L before meals and ≤6.7 mmol/L 2 h after meals. In specific cases, postprandial serum glucose levels can be measured 1 h after eating (≤7.8 mmol). Nighttime serum glucose levels should not fall below 3.3 mmol/L. Additionally, glycosylated hemoglobin (HbA1c) levels should be maintained below 5.5% during pregnancy. Pregnant women with GDM must adhere to a balanced diet and engage in regular physical activity during pregnancy.

If the goals of serum glucose levels cannot be achieved through lifestyle management, drug therapy (mainly insulin) is initiated. A total of 19 patients (4.13%) in the 100-g OGTT group and 19 patients (4.42%) in the 75-g OGTT group required medical treatment.

**1.3 Statistical Analysis**

**1.3.1 Logistic Regression Analysis**

We estimated the OR of each outcome using logistic regression models adjusted for correlated errors due to pregnancies per woman. A series of ORs were estimated using the following unadjusted and covariate-adjusted models:

1. Unadjusted analyses: group comparison only.
2. Adjusted for GDM and covariates associated with non-adherence: maternal age, BMI, pregnancy history, insulin treatment, and chronic hypertension.

**1.3.2 Variance Inflation Factor**

We performed the variance inflation factor test to check for multicollinearity. Because of the presence of multicollinearity, maternal age, BMI, pregnancy history, insulin treatment, and chronic hypertension were examined individually for their relationship with GDM.

**1.3.3 Testing for Interactions Using Log-Linear Model Analysis**

We considered several pairwise interaction terms based on the literature, including hypoalbuminemia with gestational hypertension, hypoalbuminemia with postpartum infection, neonatal hyperbilirubinemia with cesarean section, and GDM with excessive gestational weight gain as defined by the National Academy of Medicine (NAM). We found that the interaction between GDM and weight gain above NAM was statistically significant for cesarean section and gestational hypertension and was therefore included in the models for these outcomes in all comparison groups. No other pairwise interactions were statistically significant.

**2 Supplementary Tables**

**Supplementary Table 1.** Comparison of Blood Glucose Control Information in the GDM-Positive Population

| Evaluation content | 75-g OGTT  (N=430) | 100-g OGTT (N=460) | t/χ^2^ | *p* |
| --- | --- | --- | --- | --- |
| Good compliance rate [% (n/N)]^†^ | 60.93 (262/430) | 58.91 (271/460) | 0.376 | 0.584 |
| Non-maternal adverse event rate [% (n/N)]^‡^ | 1.86 (8/430) | 2.17 (10/460) | 0.110 | 0.815 |
| Insulin treatment rate [% (n/N)] * | 4.42 (19/430) | 4.13 (19/460) | 0.045 | 0.869 |
| Number of available cases of fasting blood glucose in the first month before delivery [% (n/N)] | 46.28 (199/430) | 45.87 (211/460) | 0.015 | 0.946 |
| Fasting blood glucose level within 1 month before delivery (mean±SD, mmol/L) | 4.72±0.52 | 4.73±0.53 | -0.232 | 0.817 |
| Number of available cases of HbA1c in the first month before delivery [% (n/N)] | 24.19 (104/430) | 25.00 (115/460) | 0.079 | 0.815 |
| HbA1c level within 1 month before delivery (mean±SD, %) | 5.43±0.90 | 5.43±0.70 | -0.007 | 0.994 |

t/χ^2^：Student’s *t*-test was used for continuous variables and chi-squared test was used for count data. ^†^Good compliance: patients with GDM were advised to adhere to their treatment plan, which included dietary control and regular exercise. ^‡^Non-pregnancy adverse events: these included accidental injuries, depression, pancreatitis, cholecystitis, or fractures. *Insulin treatment: insulin treatment is initiated if dietary control alone fails to achieve target blood glucose levels. Specifically, insulin should be initiated when fasting blood glucose is ≥5.3 mmol/L or 2-h postprandial blood glucose is ≥6.7 mmol/L. Regular monitoring of blood glucose levels is crucial to adjust the insulin dosage accordingly. The goal is to maintain fasting blood glucose <5.3 mmol/L and 2-h postprandial blood glucose <6.7 mmol/L, which are considered normal standards for GDM patients. GDM, gestational diabetes mellitus; GLU, glucose; HbA1c, hemoglobin A1c.

**Supplementary Table 2.** Testing for Pairwise Interaction Terms

| Interactions | *χ2* | *p* |
| --- | --- | --- |
| Hypoalbuminemia × gestational hypertension | 0.412 | 0.506 |
| Hypoalbuminemia × postpartum infection | 0.555 | 0.604 |
| Neonatal hyperbilirubinemia × cesarean section | 0.122 | 0.758 |
| GDM × weight gain beyond NAM | 3.265 | 0.021 |

GDM, gestational diabetes mellitus; NAM, National Academy of Medicine.

**Supplementary Table 3.** Association Between OGTT Screening Strategy (100g vs 75g) and Incidence of Maternal and Neonatal Complications

| Adverse Outcome | 100g OGTT Group | | 75g OGTT Group | | e（n（%）） |
| --- | --- | --- | --- | --- | --- |
|  | a（n（%）） | b（n（%）） | c（n（%）） | d（n（%）） |  |
| Abnormal fetal membranes | 237 (15.19) | 69 (16.35) | 224 (14.97) | 66 (15.35) | 946 (14.97)^Δ^ |
| Abnormal stage of labor | 10 (0.64) | 3 (0.71) | 13 (0.87) | 6 (1.40) | 55 (0.87) |
| Abnormal umbilical cord | 453 (29.04) | 121 (28.67) | 464 (31.02) | 130 (30.23) | 1330 (21.04)^Δ^ |
| Amniotic fluid volume abnormality | 69 (4.42) | 20 (4.74) | 82 (5.48) | 34 (7.91) | 400 (6.33)^Δ^ |
| Cesarean section | 578 (37.05) | 188 (44.55)* | 575 (38.44) | 205 (47.67)* | 3869 (61.21)^Δ^ |
| Cholestatic syndrome | 14 (0.90) | 6 (1.42) | 9 (0.60) | 9 (2.09) | 90 (1.42)^Δ^ |
| Dystocia | 79 (5.06) | 21 (4.98) | 81 (5.41) | 16 (3.72) | 202 (3.20)^Δ^ |
| Hypoproteinemia | 23 (1.47) | 8 (1.90) | 34 (2.27) | 9 (2.09) | 299 (4.73)^Δ^ |
| Perineal laceration | 332 (21.28) | 87 (20.62) | 342 (22.86) | 97 (22.56) | 1326 (20.98)^Δ^ |
| Pregnancy-induced hypertension | 41 (2.63) | 31 (7.35) | 37 (2.47) | 30 (6.98) | 239 (3.78)^Δ^ |
| Placental abnormalities | 55 (3.53) | 15 (3.55) | 57 (3.81) | 11 (2.56) | 309 (4.89)^Δ^ |
| Poor postpartum uterine rejuvenation | 96 (6.15) | 23 (5.45) | 84 (5.61) | 20 (4.65) | 376 (5.95)^Δ^ |
| Postpartum hemorrhage | 30 (1.92) | 12 (2.84) | 17 (1.14) | 9 (2.09) | 165 (2.61)^Δ^ |
| Postpartum infection | 12 (0.77) | 6 (1.42) | 15 (1.00) | 9 (2.09) | 59 (0.93) |
| others | 10 (0.64) | 10 (2.37) | 25 (1.67)^#^ | 11 (2.56) | 169 (2.67)^Δ^ |
| Abnormal fetal position | 324 (20.77) | 107 (25.36) | 300 (20.05) | 93 (21.63) | 1842 (29.14)^Δ^ |
| Fetal distress | 8 (0.51) | 3 (0.71) | 12 (0.80) | 5 (1.16) | 259 (4.10)^Δ^ |
| Fetal growth restriction | 16 (1.03) | 8 (1.90) | 20 (1.34) | 11 (2.56) | 80 (1.27) |
| Low birth weight | 23 (1.47) | 8 (1.90) | 31 (2.07) | 7 (1.63) | 147 (2.33)^Δ^ |
| Large for gestational age | 49 (3.14) | 10 (2.37) | 34 (2.27) | 10 (2.33) | 237 (3.75)^Δ^ |
| Low Apgar score | 7 (0.45) | 4 (0.95) | 11 (0.74) | 1 (0.23) | 51 (0.81) |
| Macrosomia | 79 (5.06) | 27 (6.40) | 95 (6.35) | 30 (6.98) | 593 (9.38)^Δ^ |
| Neonatal cranial hematoma | 96 (6.15) | 24 (5.69) | 81 (5.41) | 28 (6.51) | 562 (8.89)^Δ^ |
| Neonatal asphyxia | 6 (0.38) | 3 (0.71) | 6 (0.40) | 1 (0.23) | 37 (0.59) |
| Neonatal hyperbilirubinemia | 326 (20.90) | 80 (18.96) | 297 (19.85) | 96 (22.33) | 1907 (30.17)^Δ^ |
| Neonatal hypoglycemia | 10 (0.64) | 3 (0.71) | 12 (0.80) | 5 (1.16) | 102 (1.61)^Δ^ |
| Neonatal infection | 274 (17.56) | 77 (18.25) | 235 (15.71) | 80 (18.60) | 1633 (25.83)^Δ^ |
| Neonatal respiratory distress syndrome | 7 (0.45) | 1 (0.24) | 8 (0.53) | 6 (1.40) | 53 (0.84) |
| Preterm delivery | 49 (3.14) | 16 (3.79) | 49 (3.28) | 25 (5.81) | 361 (5.71)^Δ^ |
| Small for gestational age | 6 (0.38) | 3 (0.71) | 4 (0.27) | 1 (0.23) | 34 (0.54) |
| Stillbirth | 13 (0.83) | 5 (1.18) | 13 (0.87) | 4 (0.93) | 93 (1.47)^Δ^ |

Note: a: 100g OGTT, GDM-negative individuals (n=1,560); b: 100g OGTT, GDM-positive individuals (n=422); c: 75g OGTT, GDM-negative individuals (n=1,496); d: 75g OGTT, GDM-positive individuals (n=430); e: Individuals who did not undergo OGTT screening (n=6,321). * indicates a statistically significant difference between column a and b or between column c and d, with the symbol marked on column b or d, respectively; ^#^ indicates a statistically significant difference between column a and c or between column b and d, with the symbol marked on column c or d, respectively; ^Δ^ indicates a statistically significant difference between the screened groups (a+b+c+d) and the unscreened group e, with the symbol marked on group e.

**Supplementary Table 4.** Summary of regression parameters

| Fig.1 | Groups | Regression Equation | R2 | *p* | Slope 95%CI | F | *p* |
| --- | --- | --- | --- | --- | --- | --- | --- |
| a | 75g | y = 1.571x + 0.144 | 0.166 | ＜0.0001 | 1.416-1.727 | 1.281 | 0.258 |
|  | 100g | y = 1.698x - 0.420 | 0.195 | ＜0.0001 | 1.544-1.852 |  |  |
| b | 75g | y = 1.077x + 1.560 | 0.138 | ＜0.0001 | 0.958-1.195 | 23.280 | ＜0.0001 |
|  | 100g | y = 1.489x - 0.385 | 0.240 | ＜0.0001 | 1.370-1.607 |  |  |
| c | 75g | y = 0.461x + 3.148 | 0.377 | ＜0.0001 | 0.435-0.487 | 6.079 | 0.014 |
|  | 100g | y = 0.508x + 2.759 | 0.413 | ＜0.0001 | 0.481-0.535 |  |  |

**Note:** Data correspond to the scatter plots in Figure 1. a(fast vs 1h)；b(fast vs 2h)；c(1h vs 2h).
